# Expression Level of ADAMTS1 in Granulosa Cells of PCOS Patients Is Related to Granulosa Cell Function, Oocyte Quality, and Embryo Development

**DOI:** 10.3389/fcell.2021.647522

**Published:** 2021-04-12

**Authors:** Guang Yang, Guidong Yao, Ziwen Xu, Huiying Fan, Xingui Liu, Jiahuan He, Yue Kong, Deqi Kong, Yucheng Bai, Qina He, Tongwei Zhang, Junya Zhang, Yingpu Sun

**Affiliations:** ^1^Center for Reproductive Medicine, The First Affiliated Hospital of Zhengzhou University, Zhengzhou, China; ^2^Henan Key Laboratory of Reproduction and Genetics, The First Affiliated Hospital of Zhengzhou University, Zhengzhou, China

**Keywords:** embryo development, ADAMTS1, PCOS (polycystic ovarian syndrome), granulosa cell, oocyte quality

## Abstract

A disintegrin and metalloproteinase with thrombospondin motifs 1 (ADAMTS1) is an extracellular matrix metalloproteinase that plays an important role in the process of ovulation. According to previous studies, the expression level of ADAMTS1 in the granulosa cells of polycystic ovarian syndrome (PCOS) patients and the mechanism for regulating oocyte quality and embryonic development potential are still unclear. Our research clarified that ADAMTS1 was significantly increased in granulosa cells of PCOS patients as compared to ovulatory controls. After silencing ADAMTS1 in granulosa cells, cell proliferation and E_2_ secretion were significantly inhibited, which may be related to the down-regulation of B-cell lymphoma 2 (Bcl2) family genes and key genes involved in E_2_ synthesis. Through retrospective analysis of the clinical data, it was found that the expression level of ADAMTS1 was significantly positively correlated to the oocyte maturation rate and good-quality embryo rate in PCOS patients. The downregulation of ADAMTS1 in primary granulosa cells lead to the changes in the expression of marker genes for oocyte and embryonic quality. By using immunofluorescence staining, it was found ADAMTS1 was expressed in various stages of pre-implantation embryo but its expression level gradually decreases with the development of the embryo. In addition, the silence of ADAMTS1 in 3PN zygotes significantly prolonged the development time of the zygote to the morula stage. This is, to our knowledge, the first time to explored the mechanism by which ADAMST1 is involved in affecting the quality of oocytes and embryonic development potential, which will provide new evidence for further understanding of the follicular microenvironment and embryo development.

## Introduction

A disintegrin and metalloproteinase with thrombospondin motifs (ADAMTS) is a family of extracellular proteases consisting of 19 mammalian members (Dubail and Apte, 2015). These members contain multiple domains such as a signal peptide, a metalloproteinase domain, a disintegrin-like domain, and a thrombospondin type1 sequence repeat (TSR) motif. Previous studies have shown that ADAMTS family members play a major role in organogenesis, and are also associated with physiological processes, such as angiogenesis, wound healing, and tumor cell migration (Kelwick et al., 2015). Among these members, ADAMTS1 is vital for the reproductive system, especially in the regulation of ovarian function.

ADAMTS1 was first reported in 1997 and found to be localized on chromosome 21q21.3, encoding a protein of 591 amino acids (Kuno et al., 1997). It is a secreted protein that can be activated by furin protease and then anchored to the extracellular matrix (ECM) based on the specificity of TSR and carboxy-terminal spacer domain, whereby exerting biological functions (Porter et al., 2005). ADAMTS1 is widely expressed in many organs, such as ovary, placenta, gall bladder, and prostate. In addition, it participates in several pathophysiological processes, such as inflammation, atherosclerosis, pulmonary fibrosis, and cancer cell invasion (Qu and Zhang, 2018; Eissa et al., 2019). Notably, ADAMTS1 is highly expressed in the ovary. Previous studies also demonstrated that ADAMTS1 participates in the morphogenesis and function maintenance of female reproductive organs through the dynamic shaping of ECM (Shindo et al., 2000). In addition, it also plays a major role in regulating follicle development and gametogenesis. Before ovulation, ADAMTS1 is strongly expressed in the dominant follicles, and rapidly degrades ECM and ruptures the follicle wall, thereby promoting ovulation (Brown et al., 2006). The abnormal expression of ADAMTS1 can lead to the failure of ovulation and fertilization. The knockout of ADAMTS1 in mice limited the expansion of cumulus-oocyte complex (COCs), trapped the oocytes in the follicle wall, and significantly reduced the rates of ovulation and fertilization (Brown et al., 2010). These phenomena could be attributed to the abnormal localization and expression of many key components of ECM, which are the substrates of ADAMTS1. In addition, some studies have found that ADAMTS1 is also associated with a variety of clinical ovarian diseases, such as premature ovary failure (POF) (Yao et al., 2019) and ovarian cancer (Liu et al., 2015). Therefore, it is speculated that ADAMTS1 is closely related to ovarian function.

Polycystic ovarian syndrome (PCOS) is one of the common causes of female infertility and is accompanied by hormonal imbalance and metabolism issues, such as hyperandrogenism (HA), obesity, insulin resistance (IR), and hyperinsulinemia (HI) (Rosenfield and Ehrmann, 2016). These factors lead to small follicle aggregation and polycystic changes in the ovaries, resulting in oligo-ovulation/anovulation. In addition, low-grade chronic inflammation, oxygen stress, and mitochondrial dysfunction are considered as the pathogenesis of PCOS (Duleba and Dokras, 2012; De Leo et al., 2016; Papalou et al., 2016). Furthermore, PCOS patients undergoing *in vitro* fertilization (IVF) or intracytoplasmic sperm injection (ICSI) are often accompanied by the clinical characteristics, such as high oocyte retrieval number and high incidence rate of ovarian hyperstimulation syndrome (OHSS). It is also speculated that the oocyte maturation rate and the fertilization rate of PCOS patients are lower than those of the ovulatory controls (Patel and Carr, 2008). However, due to the diversity of clinical features of PCOS and with the improvement in human-assisted reproductive technologies, there are currently different opinions on the clinical outcome of PCOS patients receiving IVF/ICSI-ET (Siristatidis et al., 2015).

According to previous studies, ADAMTS1 was found to be elevated in the follicular fluid and blood of PCOS patients (Tola et al., 2017, 2018), but down-regulated in granulosa cells (Xiao et al., 2014). It was also found that the expression of ADAMTS1 may affect the quality of oocytes and the potential of embryonic development. However, these studies only conducted a correlation analysis, and none of them were focused on the underlying mechanisms which is the purpose of this work.

## Materials and Methods

### Sample Collection

This study was approved by the Biomedical Ethics Committee of the First Affiliated Hospital of Zhengzhou University, and written informant consent was obtained from the patients.

Of the total 92 patients who were received IVF or ICSI treatments in Center for Reproductive Medicine of the First Affiliated Hospital of Zhengzhou University, 48 patients were diagnosed with PCOS according to the 2008 Rotterdam criteria, and the remaining 44 patients constituted the control group. The control group referred to the female patient with normal ovulation cycle but is infertile due to the tubal factor or male factors. Patients with ovarian dysfunction, endometriosis, chromosomal abnormalities, and other systemic diseases were excluded from the study cohort. The clinical data used in this study was derived from the clinical reproductive system in our center. The baseline endocrine hormones were measured at 2–3 days of menstruation.

IVF-ET laboratory indicators: oocyte maturation rate (%) = (number of metaphase II (MII) oocytes/number of oocytes retrieved) × 100, fertilization rate (%) = (number of fertilized oocytes/number of oocytes retrieved) × 100, cleavage rate (%) = (number of cleaved embryos/number of fertilized oocytes) × 100, good-quality embryo rate (%) = (number of good-quality embryos/number of cleaved embryos) × 100, morula formation rate (%) = (number of morulae/number of embryos cultured) × 100, blastocyst formation rate (%) = (number of blastocysts/number of embryos cultured) × 100, and good-quality blastocyst rate (%) = (number of good-quality blastocysts/number of blastocysts) × 100.

### Human Primary Granulosa Cell Extraction and Cell Culture

The primary granulosa cells were purified by density gradient centrifugation from the follicular fluid collected on the day of oocyte retrieval, as described previously (Chang et al., 2013). KGN, a human granulosa-like tumor cell line, was a gift from Prof. Fei Sun (University of Nantong, Nantong, China). Both primary granulosa cells and KGN cell line were cultured in DMEM/F12 (Thermo Fisher Scientific, Waltham, United States) medium supplemented with 10% charcoal/dextran-treated FBS (HyClone, Logan, United States), 100 U/mL of penicillin and 100 μg/mL of streptomycin sulfate (HyClone) in a humidified atmosphere of 95% air and 5% CO_2_ at 37°C.

### Embryo Culture and Quality Evaluation

The clinically discarded 3PN zygotes (day 1) were collected for siRNA injection and cultured in GERI timelapse imaging system (Genea biomedx, Sydney, Australia). After injection, zygotes were cultured in G-1 Plus (Vitrolife, Goteborg, Sweden) medium covered with mineral oil to day 3. Then the cleaved embryos were transferred to G-2 Plus (Vitrolife, Goteborg, Sweden) medium covered with mineral oil till day 5/6. The embryo quality was evaluated according to the Peter scoring system on day 3 (Brinsden, 1999), and blastocyst formation was evaluated by using Gardner blastocyst scoring system on day 5/6 (Gardner et al., 2000).

### Immunofluorescence

Primary granulosa cells were fixed for 20 min in 4% paraformaldehyde at room temperature and permeabilized with 0.05% Triton X-100 in phosphate-buffered saline (PBS) for 20 min, followed by blocking with 5% BSA in PBS for 30 min and probed with ADAMTS1 (Abcam, Cambridge, United States) or Ki-67 (Cell Signaling Technology, Beverly, United States) primary antibody at 4°C overnight. Subsequently, the samples were incubated with secondary anti-rat antibody (Jackson ImmunoResearch, West Grove, United States) for 40 min and stained with DAPI (Sigma, St Louis, United States) for 15 min before acquiring images using a Zeiss microscope (Carl Zeiss, Oberkochen, Germany).

### Real-Time PCR

TRIzol reagent (Invitrogen, Carlsbad, United States) was used to extract the total RNA from the primary granulosa cells according to the manufacturer’s protocol, and the RNA concentration was measured by NanoDrop (Thermo Fisher Scientific). RNA was reverse transcribed into cDNA using the RT Kit (Bio-Rad Laboratories, Hercules, United States), and real-time PCR was performed on a 7500 Real-Time PCR System (Bio-Rad Laboratories) using 5 × SYBR Green SuperMix (Bio-Rad Laboratories). The method of 2^–△^^△^^Ct^ was used to calculate the relative expression of the target gene as compared to that of the internal reference: △Ct = △Ct_*target*_ – △Ct_*reference*_, – △△Ct = – (△Ct_*treat*_ – △Ct_*control*_). The sequences of the primers (Invitrogen) are shown in the Supplementary Table 1. The melting curve was used to verify the primer specificity. GAPDH was used as an internal reference gene.

### RNA Transfection

On the day before transfection, the primary granulosa cells or the KGN cells were seeded in 6-well plates. When the cell density reached 70% confluency, the culture medium was replaced by DMEM/F12 only medium, and the transfection reagent mixture containing Opti-MEM (Thermo Fisher Scientific), lipofectamine 2000 (Invitrogen) and ADAMTS1/control siRNA (Invitrogen, catalog#1299001/12935400) was added dropwise into each well. After 6 h, the culture medium was replaced with the normal culture medium, and the cells and/or the supernatant were collected after culturing for an additional 48 h.

### Western Blot

The primary granulosa cells were lysed using cell lysis buffer (Sangon Biotech, Shanghai, China). Equivalent amounts of protein were separated by sodium dodecyl sulfate polyacrylamide gel electrophoresis (SDS-PAGE) and transferred to a polyvinylidene difluoride (PVDF) membrane. After blocking in 5% non-fat dry milk in tris-buffered saline (TBS) for 1 h, the membranes were probed with primary antibodies against ADAMTS1 (Abcam), Bcl-2 (Abcam), Bcl-XL (Abcam) and Bax (Proteintech, Wuhan, China) at 4°C overnight. On the following day, the membranes were incubated with the appropriate HRP-conjugated secondary antibody. The immunoreactive bands were detected using an enhanced chemiluminescent substrate (Bio-Rad Laboratories). The ChemiDoc MP Imager (Bio-Rad Laboratories) was used to acquire the images of the chemiluminescent blots.

### Cell Proliferation Analysis

Cell proliferation was analyzed using the CCK-8 assay kit (Boster Biological Technology, Wuhan, China). KGN cells were plated in 96-well plates at a density of 5 × 10^4^/well, and the cells were transfected with 50 nM siRNA on the next day. Subsequently, after replacing the culture medium with 100 μl fresh culture medium and 10 μl CCK-8 solution at the indicated time, the cells were cultured for another 2 h, and the OD was measured at 450 nm.

### Estradiol Measurement

In order to measure the E_2_ concentration in culture medium, the primary granulosa cells were seeded in 6-well plates at a density of 1 × 10^6^ cells/well and cultured for 4–5 days before the transfection with 50 nM siRNA. The testosterone (T) (10^–7^ mol/L) was added into the new culture medium as a substrate of the primary granulosa cells at 6 h post-transfection, and the supernatant was collected after 48 h. The E_2_ was measured by Electrochemiluminescence Immunoassay Kit (Roche Diagnostics, Rotkreuz, Switzerland) in Roche Diagnostics Cobas 6000 (Roche Diagnostics).

### Statistical Analysis

Data were analyzed using the SPSS version 19.0 statistical program. The continuous variables distributed normally were represented as mean ± standard deviation (SD). When two groups were compared, the Student’s *t*-test was used for parametric data and the Mann–Whitney *U* test was used for non-parametric data. Multiple comparisons were analyzed using a one-way ANOVA or two-way repeated measures ANOVA. Categorical comparisons were performed using the chi-square test. Univariate analysis of correlations was performed using the Spearman’s test. *p* < 0.05 was considered statistically significant.

## Results

### ADAMTS1 Is Significantly Up-Regulated in Granulosa Cells of Patients With PCOS as Compared to Ovulatory Controls

A total of 92 patients were enrolled in this study, including 44 ovulatory controls and 48 PCOS patients. The analysis of the clinical characteristics between the two groups showed that there were no differences in age, prolactin (PRL), E_2_, and total gonadotropin (Gn) dose (Table 1). However, patients in the PCOS group had significantly higher BMI (*p* = 0.0005), anti-Mullerian hormone (AMH) (*p* = 0.0001), luteinizing hormone (LH) (*p* = 0.0043), and T (*p* < 0.0001) than that in the control group, while the level of follicle stimulating hormone (FSH) was significantly lower than that of the control group (*p* = 0.011) (Table 1).

**TABLE 1:** Clinical characteristics of the enrolled patients.

	**Normal (*n* = 44)**	**PCOS (*n* = 48)**	***P*-value**
Age (year)	30.09 ± 0.73	29.52 ± 0.54	0.5278
BMI (kg/m^2^)	22.66 ± 0.45	24.86 ± 0.42	0.0005
AMH (ng/ml)	3.93 ± 0.45	7.18 ± 0.66	0.0001
FSH (IU/L)	6.98 ± 0.33	6.00 ± 0.20	0.011
LH (IU/L) E_2_ (pg/ml)	6.14 ± 0.64 40.16 ± 2.56	9.68 ± 0.99 47.39 ± 3.81	0.0043 0.1258
T (ng/mL)	0.29 ± 0.02	0.47 ± 0.03	<0.0001
P(ng/ml)	0.43 ± 0.05	0.47 ± 0.09	0.7548
PRL (ng/ml)	19.42 ± 1.62	17.68 ± 1.81	0.4769
Total Gn dose (IU)	2363 ± 142.50	2152 ± 98.69	0.2186

*BMI, body mass index; AMH, anti-mullerian hormone; FSH, follicle stimulating hormone; LH, luteinizing hormone; E_2_, estradiol; T, testosterone; P, progesterone; PRL, prolactin; Gn, gonadotropin. *p* < 0.05 was considered statistically significant.*

**FIGURE 1:**
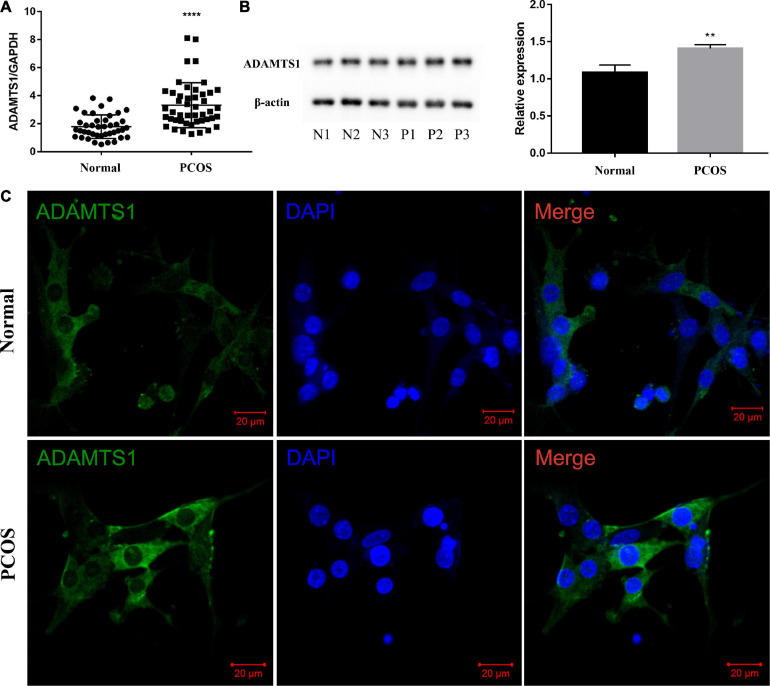
ADAMTS1 is up-regulated in granulosa cells of PCOS patients. Real-time PCR analysis of ADAMTS1 mRNA expression in primary granulosa cells from control and PCOS patients **(A)**. GAPDH was used as an internal control. **** Indicates *p* < 0.0001. The protein levels of ADAMTS1 detected by western blot in primary granulosa cells were from control and PCOS patients **(B)**. The immunofluorescence staining was used to detect the expression and localization of ADAMTS1 in granulosa cells of ovulatory controls and PCOS patients **(C)**. Green, ADAMTS1. Blue, DAPI indicates the nuclear localization signal. Bar = 20 μm.

The estimation of the mRNA (Figure 1A) and protein level (Figure 1B) of ADAMTS1 in the human primary granulosa cells between the two groups revealed that the level of ADAMTS1 was significantly higher in the PCOS patients than the ovulatory controls (*p* < 0.0001). Furthermore, an immunofluorescence assay was conducted to investigate the expression and localization of ADAMTS1 in granulosa cells, and the results showed that ADAMTS1 was mainly expressed in the cytoplasm of granulosa cells, and the staining intensity of ADAMTS1 in granulosa cells from PCOS patients was stronger as compared with the ovulatory controls (Figure 1C).

### Granulosa Cell Function Can Be Affected by Silencing ADAMTS1

**FIGURE 2:**
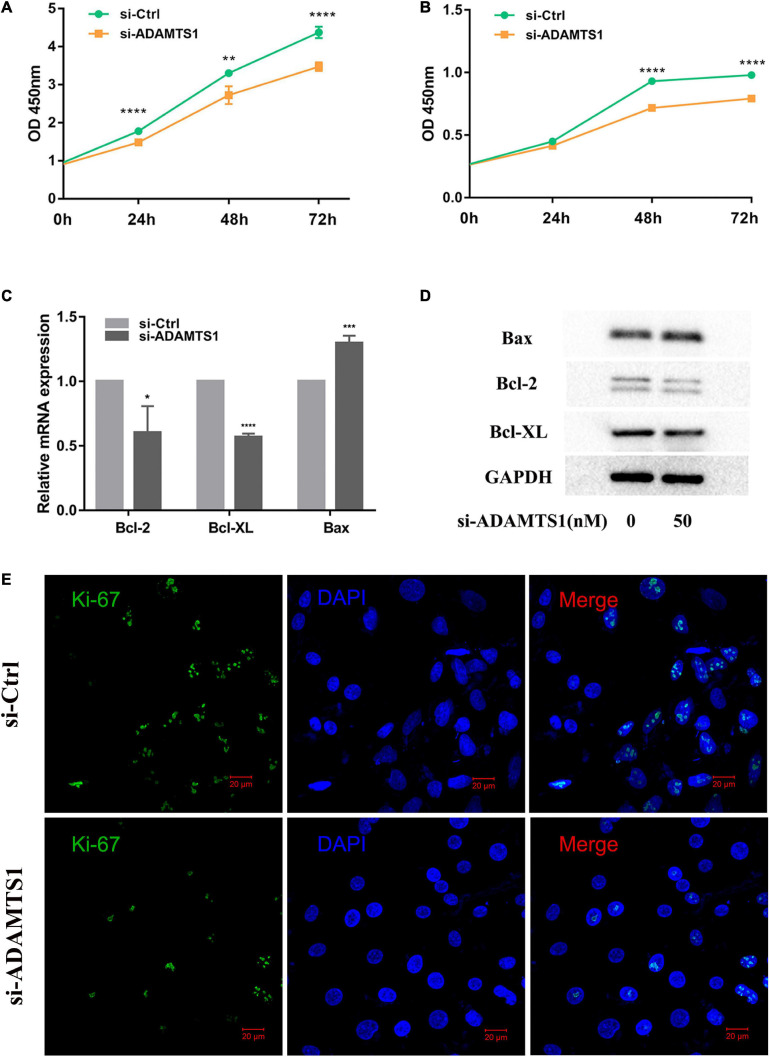
Down-regulation of ADAMTS1 inhibits proliferation in granulosa cell. Cell proliferation was measured by using CCK-8 kit in KGN **(A)** and primary granulosa cells **(B)** at 0, 24, 48, and 72 h. Primary granulosa cells were transfected with siRNA at the concentration of 50 nM for 48 h, and the mRNA **(C)** and protein levels **(D)** of Bcl-2, Bcl-XL, and Bax were analyzed. The Ki-67 staining was performed after transfection of si-ADAMTS1 to evaluate the cell proliferation **(E)**. GAPDH gene was used as an internal control. * Indicates *p* < 0.05, ** indicates *p* < 0.01, *** indicates *p* < 0.001, and **** indicates *p* < 0.0001.

Then, we speculated that ADAMTS1 might affect the function of granulosa cells. After we silenced ADAMTS1, the mRNA and protein level of ADAMTS1 were significantly down-regulated (Supplementary Figure 1). Cell proliferation was analyzed both in KGN (Figure 2A) and primary granulosa cells (Figure 2B) by using the CCK-8 Kit, and the results showed that granulosa cell proliferation was significantly inhibited after silencing ADAMTS1, and the difference was enhanced as the culture time was extended. In addition, the proportion of Ki-67 positive cells in the si-ADAMTS1 treatment group was significantly reduced (Figure 2E). Cell proliferation and apoptosis-related genes were also analyzed, and the results indicated that the expression of Bcl-2 and Bcl-XL was down-regulated after the silence of ADAMTS1 in primary granulosa cells, while the expression of Bax was up-regulated (Figures 2C,D).

**FIGURE 3:**
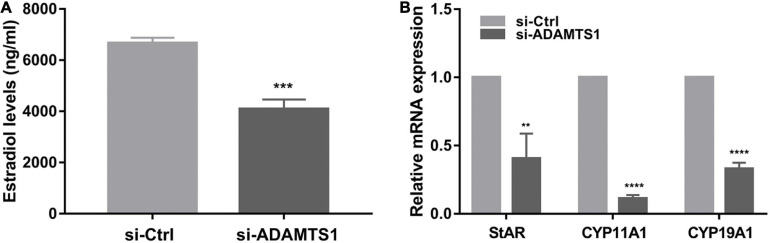
Down-regulation of ADAMTS1 inhibits estradiol secretion in granulosa cell. The E_2_ secretion in primary granulosa cells after silencing of ADAMTS1 using 50 nM si-ADAMTS1 for 48 h **(A)**. Real-time PCR analysis of the expression of StAR, CYP11A1 and CYP19A1 in primary granulosa cells after silencing of ADAMTS1 by 50 nM si-ADAMTS1 for 48 h **(B)**. GAPDH gene was used as an internal control. ** Indicates *p* < 0.01, *** indicates *p* < 0.001, and **** indicates *p* < 0.0001.

In addition, E_2_ was analyzed in primary granulosa cells. The results showed that the level of E_2_ in culture medium was significantly down-regulated after the silence of ADAMTS1 as compared to the control group (*p* < 0.001) (Figure 3A). This phenomenon could be attributed to the down-regulation of the expression of StAR, CYP11A1, and CYP19A1 genes (Figure 3B).

### Expression of ADAMTS1 in Granulosa Cells Is Associated With the Embryo Quality

After evaluating the function of ADAMTS1 on the granulosa cells, we further explored the effect on oocyte quality and early embryonic development. The laboratory data of the embryos between ovulatory controls and PCOS patients were compared firstly. As the results shown that there was no significant difference in the number of oocytes retrieved, oocyte maturation rate, fertilization rate, and cleavage rate between the two groups (*p* > 0.05). However, the rate of good-quality embryo and blastocyst formation was significantly lower in the PCOS group than that in the control group (*p* < 0.05) (Table 2).

**TABLE 2:** IVF-ET laboratory indicators in normal and PCOS patients.

	**Normal (*n* = 44)**	**PCOS (*n* = 48)**	***p-*value**
Oocytes retrieved (*n*)	14.89 ± 0.78	16.06 ± 0.82	0.304
Oocyte maturation rate (%)	81.8	78.1	0.079
Fertilization rate (%)	61.9	64.3	0.348
Cleavage rate (%)	99.5	98.5	0.185
Good-quality embryo rate (%)	70.6	59.8	0.000
Blastocyst formation rate (%)	62.2	52.6	0.026

*Oocyte maturation rate (%) = (number of MII oocytes/number of oocytes retrieved) × 100; Fertilization rate (%) = (number of fertilized oocytes/number of oocytes retrieved) × 100; Cleavage rate (%) = (number of cleaved embryos/number of fertilized oocytes) × 100; Good-quality embryo rate (%) = (number of good-quality embryos/number of cleaved embryos) × 100; Blastocyst formation rate (%) = (number of blastocysts/number of embryos cultured) × 100. *p* < 0.05 was considered statistically significant.*

**FIGURE 4:**
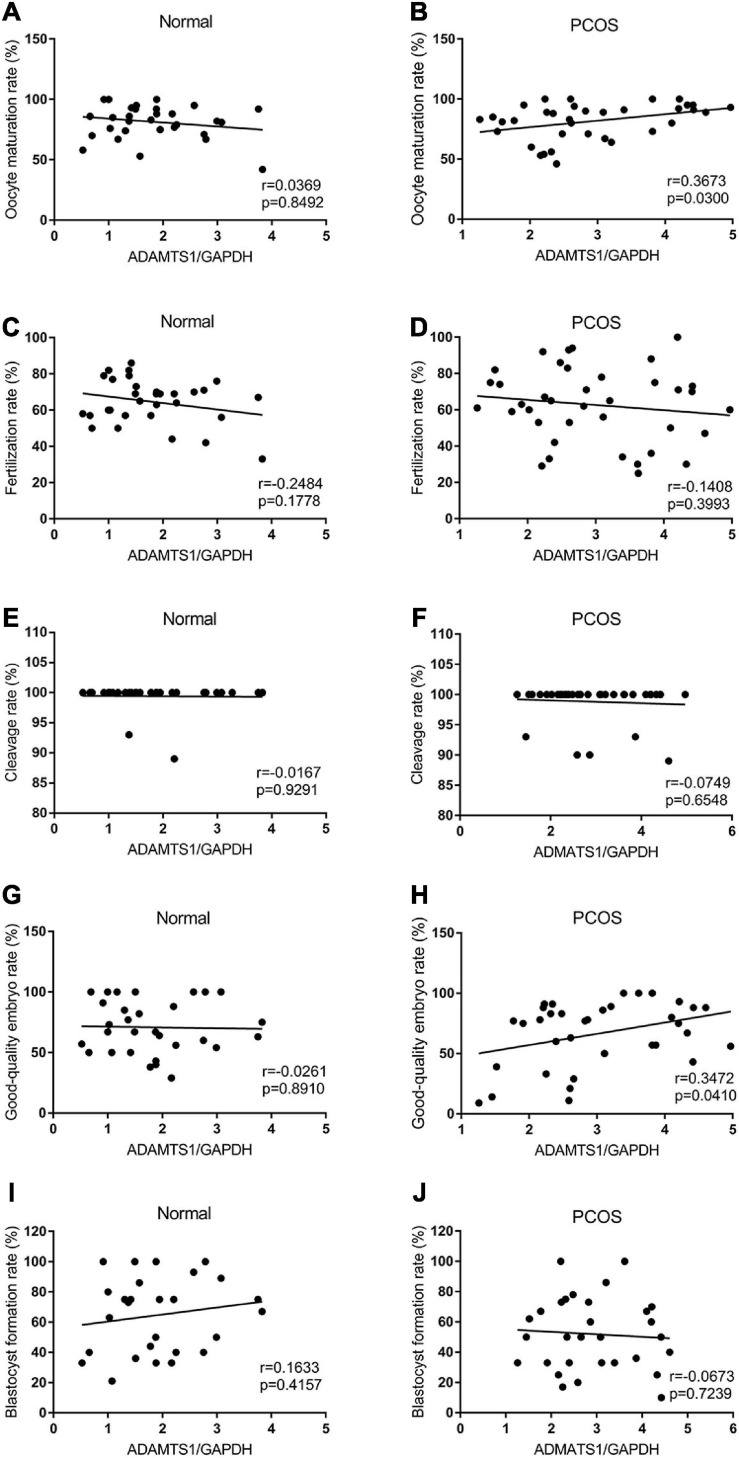
Scatter plots were used to depicting the correlation between the IVF-ET laboratory indicators including oocyte maturation rate **(A,B)**, fertilization rate **(C,D)**, cleavage rate **(E,F)**, good-quality embryo rate **(G,H)** and blastocyst formation rate **(I,J)** and the expression of ADAMTS1 in granulosa cells from control and PCOS patients. Statistical analysis of the data was performed using the Spearman test.

Also, the correlation between ADAMTS1 expression and IVF-ET laboratory indicators was analyzed. The results showed the expression of ADAMTS1 was not associated with oocyte maturation rate (Figure 4A), fertilization rate (Figure 4C), cleavage rate (Figure 4E), good-quality embryo rate (Figure 4G), and blastocyst formation rate (Figure 4I) in the control group (*p* > 0.05). However, it was positively correlated with the oocyte maturation rate (Figure 4B) and the good-quality embryo rate (Figure 4H) (*p* < 0.05) in the PCOS group, but not related to the fertilization rate (Figure 4D), cleavage rate (Figure 4F) and blastocyst formation rate (Figure 4J) (*p* > 0.05).

### Oocyte- and Embryonic Development-Related Genes Can Be Regulated by ADAMTS1 in Granulosa Cells

**FIGURE 5:**
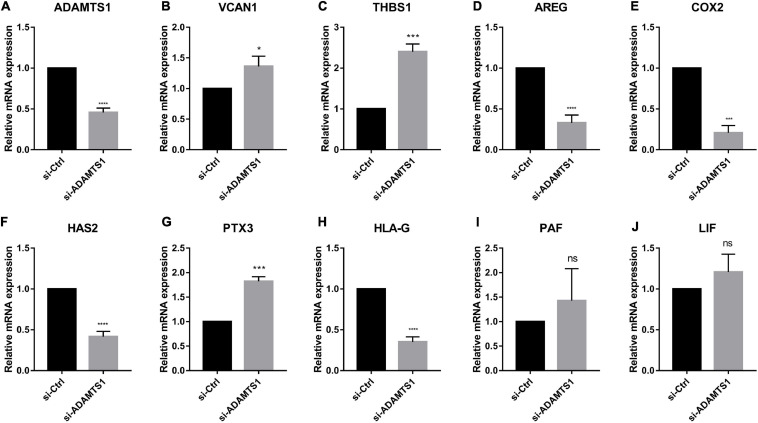
Embryonic development related genes can be regulated after silencing of ADAMTS1 **(A)** in granulosa cells. The relative expression of VCAN1 **(B)**, THBS1 **(C)**, AREG **(D)**, COX2 **(E)**, HAS2 **(F)**, PTX3 **(G)**, HLA-G **(H)**, PAF **(I)**, and LIF **(J)** in primary granulosa cells after silencing ADAMTS1 gene by 50 nM siRNA. GAPDH gene was used as an internal control. * Indicates *p* < 0.05, *** indicates *p* < 0.001, **** indicates *p* < 0.0001 and “ns” indicates no significant difference.

Similarly, we down-regulated ADAMTS1 in primary granulosa cells and evaluated the expression of oocyte quality marker genes (Figure 5). The result confirmed that the level of VCAN1, which is target substrate of ADAMTS1 protein, was increased significantly in granulosa cells after silencing ADAMTS1 (*p* < 0.05). In addition, the expression of PTX3 and THBS1 was increased, while the expression of AREG, HAS2, COX2, and HLA-G was decreased significantly (*p* < 0.05); however, no obvious changes were detected in LIF and PAF (*p* > 0.05). These findings implied that ADAMTS1 could alter the expression of embryonic development-related genes, thereby affecting the quality of oocytes and subsequent embryonic development.

### Silence of ADAMTS1 in Zygotes Affects Embryonic Developmental Timing

**FIGURE 6:**
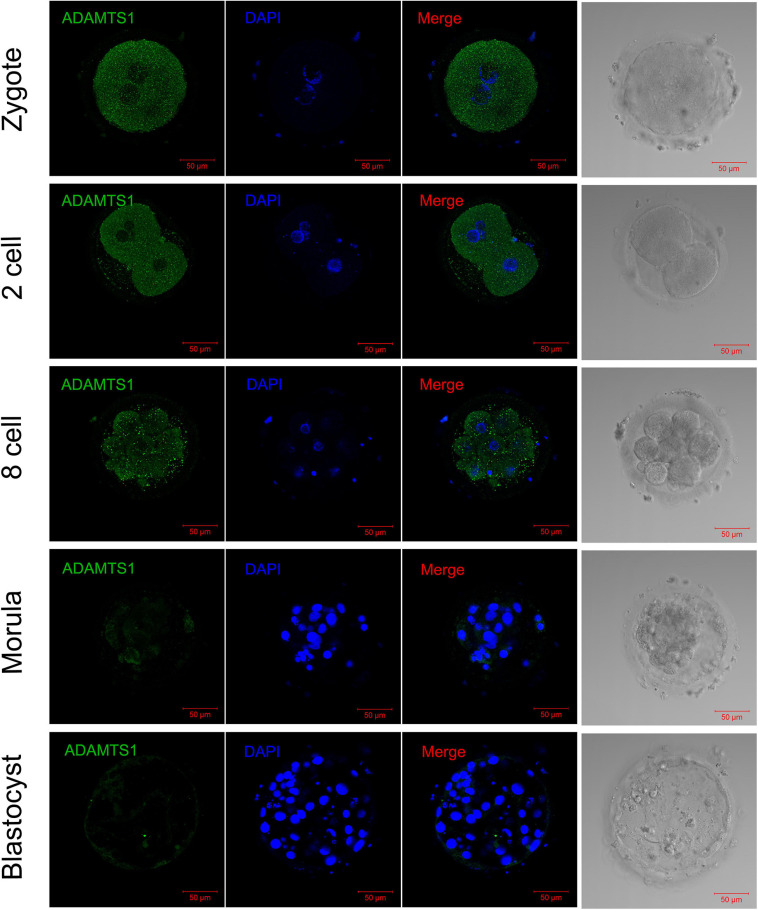
The expression and localization of ADAMTS1 in various stages of embryos. Immunofluorescence of ADAMTS1 in human zygote, two cell embryos, eight cell embryos, morula and blastocyst, the control staining without ADAMTS1 antibody. Green, ADAMTS1. Blue, DAPI indicates the nuclear localization signal. Bar = 50 μm.

In order to explore the impact of ADAMTS1 on the early embryonic development, we then observed its expression in embryos. The immunofluorescence staining revealed that ADAMTS1 was expressed in various stages of pre-implantation embryonic development, and mainly localized in the cytoplasm (Figure 6). The staining intensity weakened gradually with the development of the embryo.

By analyzing the results of embryo development after silencing ADAMTS1 in clinically discarded 3PN zygotes, it was found that the development time of zygotes to morula stage was significantly prolonged (*p* < 0.05). There were no significant differences in good-quality embryo rate, morula formation rate, blastocyst formation rate and good-quality blastocyst rate (*p* > 0.05) (Table 3).

**TABLE 3:** Studies of embryo development after silencing of ADAMTS1.

	**si-Ctrl (*n* = 48)**	**si-ADAMTS1 (*n* = 48)**	***p-*value**
Development time to morula stage (h)	68.21 ± 2.66	77.05 ± 3.09	0.037
Good-quality embryo rate (%)	76.3	65.8	0.312
Morula formation rate (%)	47.4	47.4	1.000
Blastocyst formation rate (%)	28.9	31.6	0.803
Good-quality blastocyst rate (%)	27.3	25.0	0.901

*The development time to morula stage is calculated from the time of siRNA injection. Good-quality embryo rate (%) = (number of D3 good-quality embryos/number of cleaved embryos) × 100; Morula formation rate (%) = (number of morulae/number of embryos cultured) × 100; Blastocyst formation rate (%) = (number of blastocysts/number of embryos cultured) × 100; Good-quality blastocyst rate (%) = (number of good-quality blastocysts/number of blastocysts) × 100. *p* < 0.05 was considered statistically significant.*

## Discussion

PCOS patients have abnormal endocrine characteristics and follicular microenvironment, which affects the development of oocytes. A majority of studies suggested that the quality of oocytes in PCOS patients is significantly lower than those in patients with normal ovulation cycles (Palomba et al., 2017), which was consistent with our results. However, other studies have shown that the fertilization competence and the clinical pregnancy outcomes did not significantly differ between PCOS patients and controls (Heijnen et al., 2006; Duan et al., 2018). Many factors affect the oocyte quality of PCOS patients. Some of them play the positive role, while others are the destructive one, and the combined effect of these multiple factors determines the development potential of oocytes and embryos. Hence, the mechanisms of that remain to be elucidated. The purpose of this study was to explore the underlying mechanisms that affect the quality of oocytes in patients with PCOS.

Tola et al. (2017) demonstrated that ADAMTS1 was elevated in follicular fluid and blood of PCOS patients (Tola et al., 2018). In this study, we suggested that both mRNA and protein of ADAMTS1 were up-regulated in granulosa cells of PCOS. Since the matured form of ADAMTS1 is mainly secreted by granulosa cells into the follicular fluid (Russell et al., 2003), our conclusion may be an important supplement to Tola et al. (2017, 2018) and provides a clue to the origin of ADAMTS1 in follicular fluid. Studies also shown that ADAMTS1 was reduced in cumulus cells in patients with PCOS (GohariTaban et al., 2019; Ma et al., 2020). Mural granulosa cells and cumulus cells are formed by the differentiation of granulosa cells, and they have different functional roles in follicular development, especially in the late stage. The mainly functions of mural granulosa cells are cell signaling and endocrine function, while the cumulus cells mostly communicate with oocytes via gap junctions for metabolism and regulation of meiosis. In addition, both cell types experienced dramatic transcriptomic changes, and the differences increased as the follicles grew (Wigglesworth et al., 2015). Therefore, the function and expression pattern of ADAMTS1 may be different in cumulus cells and mural granulosa cells, and whether ADAMST1 from different parts of ovary have synergistic effects on oocyte development still needs to be further studied.

According to Xiao et al. (2014), the authors suggested that the expression of ADAMTS1 was down-regulated in granulosa cells from PCOS patients as compared to the control group by using real-time PCR, and the protein was abundantly expressed both in the nucleus and cytoplasm by using immunocytochemistry. However, we found that the mRNA and protein of ADAMTS1 were up-regulated in PCOS-derived granulosa cells. By analyzing the results of immunofluorescence experiment, we can clearly see the localization signal of ADAMTS1 was mainly detected in the cytoplasm, with almost no expression in the cell nucleus. These differences may be due to the sample sizes and experimental conditions. Nonetheless, there are studies did not show any significant difference in the expression of ADAMTS1 between the two types of patients (Karakose et al., 2016; Özler et al., 2017), which might be due to the detection methods and ethnicity of the patients.

Since the function of granulosa cells is closely related to the maturation of oocytes and follicular status, and the abnormal development of oocytes is often accompanied by abnormal function of the granulosa cells. According to previous studies, the lack of mature ADAMTS1 protein may lead to oocyte developmental arrest, which might be related to abnormal proliferation of granulosa cells (Meng et al., 2017). The lower expression of ADAMTS1 may result in the inhibition of cell proliferation in some cell lines, such as HCASMCs and VSMC31 (Li et al., 2017; Qu and Zhang, 2018); however, no relevant studies have yet been carried out in granulosa cells. Herein, CCK8 and Ki-67 staining which is the marker of cell proliferation showed that the down-regulation of ADAMTS1 significantly inhibited the proliferation of primary granulosa cells, and the same result of CCK8 was obtained in the down-regulation of ADAMTS1 in the KGN cell line, which has better proliferation ability. More interestingly, we found it also lead to the down-regulation of Bcl-2 and Bcl-XL and up-regulation of Bax. Bcl-2 or Bcl-XL mainly forms a homodimer or heterodimer with Bax to exert physiological effects (Antonsson et al., 1997). The ratio of the amount of protein is considered as a “molecular switch” of cell apoptosis. Therefore, we hypothesize that ADAMTS1 affect the apoptosis of granulosa cells through the mitochondrial pathway by affecting the relative ratio of Bcl2 or Bcl-XL to Bax.

In addition, the level of hormone status in follicles markedly affects the development of oocytes, and adequate secretion of E_2_ by granulosa cells is essential for oocyte maturation (Tesarik and Mendoza, 1995). Both StAR and CYP11A1 are the key genes in the rate-limiting step of steroidogenesis, and CYP19A1 plays a major role in the conversion of testosterone into E_2_ (Andersen and Ezcurra, 2014). In this study, the E_2_ secretion can be significantly reduced by silencing ADAMTS1 in granulosa cells, which may be related to the down-regulation of StAR, CYP11A1 and CYP19A1.

Importantly, the expression level of ADAMTS1 is shown to be associated with the oocyte quality in PCOS patients (Huang et al., 2013; GohariTaban et al., 2019). It has also been confirmed that ADAMTS1 and its substrate, cleaved VCAN1, dramatically increase during COC maturation from the germinal vesicle (GV) to MII stage (Bo and Julang, 2018), which suggests a key role of ADAMTS1 in the maturation of oocytes. Our results also showed that the expression of ADAMTS1 was positively correlated with oocyte maturation rate and good-quality embryo rate in PCOS patients. Thus, we hypothesize that ADAMTS1 exerts a protective role in the development of oocyte and embryos in PCOS patients.

In order to explore the effect of the expression of ADAMTS1 on the development of oocytes and embryos, we further investigated the expression of ECM-related genes and other marker genes of oocyte and embryo quality by silencing ADAMTS1 in primary granulosa cells. As a result, the expression of AREG, HAS2, COX2, and HLA-G was significantly reduced, while that of PTX3 was up-regulated. This result further confirmed the effect of ADAMTS1 on oocyte quality and embryo development potential and that it could alter the expression of other cytokines and indirectly affect the oocyte development through paracrine pathway in the ovary.

Previous studies have shown that the expression of AREG in granulosa cells is positively correlated to the oocyte retrieval rate, oocyte maturation rate, fertilization rate, and good-quality embryo rate (Huang et al., 2015), and adding AREG during IVM significantly increases the oocyte maturation rate and embryo development potential in PCOS patients (Sanchez et al., 2017). The PTX3 expression is decreased in the serum of PCOS patients, and the reduced PTX3 may balance immune cell types to promote follicle development of PCOS patients (Camaioni et al., 2018). HAS2, a hyaluronic acid synthase, directly affects the synthesis of hyaluronic acid. Studies have reported that the expression levels of PTX3 and HAS2 in good-quality embryonic COCs are significantly higher than those of inferior embryos and unfertilized oocytes (Cillo et al., 2007), and the differential expression of PTX3 may serve as a discriminative indicator of embryo implantation potential (Zhang et al., 2005). Strikingly, COX2 and HLA-G play a critical role in the expansion of COCs and can be used as markers for the embryonic development (McKenzie et al., 2004). In this study, we also found that the expression of VCAN1 could be regulated by silencing ADAMTS1. VCAN is a large extracellular matrix proteoglycan, which has 4 splicing isomers (VCAN0, VCAN1, VCAN2, and VCAN3), arising from alternative splicing. Among them, VCAN1 is widely expressed, especially in the reproductive system and embryos (Foulcer et al., 2014), and Sandy et al. (2001) noticed a preference for ADAMTS cleavage site in VCAN1. By degrading VCAN1, ADAMTS1 promotes follicular development, especially in the expansion process of COCs (Espey et al., 2000).

More importantly, we explored the expression patterns of ADAMTS1 in embryos at various developmental stages and revealed its direct impact on embryo development for the first time. When ADAMTS1 was silenced in zygotes, the development time to morula stage was significantly prolonged. In recent years, studies have shown that the time parameters are important in the process of entire embryonic development and could be used to predict embryonic potential. Conaghan et al. and Desai et al. have shown that embryos that cleavage earlier have higher development potential (Conaghan et al., 2013; Desai et al., 2014). In addition, the rate of D3 good-quality embryos and D5 good-quality blastocyst rates both decreased after the down-regulation of ADAMTS1, which is consistent with the conclusion of that in granulosa cells. However, perhaps because the number of sample cases was small, no statistical difference was obtained. Thus, it can be hypothesized that ADAMTS1 exerts a protective role during the development of oocytes and embryos and could be used as a marker for evaluating the quality of the embryo.

This study has some limitations. First of all, we only down-regulated ADAMTS1 in granulosa cells and embryos. Since we believe that the expression of ADAMTS1 is up-regulated in PCOS patients, over-expression cell models will be more intuitive, which will be further improved in our follow-up research. Secondly, due to the small sample size, some analyses may not yield statistically differences.

Based on the above results, we concluded that ADAMTS1 could affect the function of granulosa cells, and the process of early embryo development, which may be a potential mechanism that affects the oocyte quality and embryonic potential of PCOS patients. This study explored the mechanism by which ADAMST1 is involved in affecting the quality of oocytes and embryonic development potential in PCOS patients. These results support the conclusions reported previously and provide new evidence for further understanding of the follicular microenvironment and embryo development.

## Data Availability Statement

The raw data supporting the conclusions of this article will be made available by the authors, without undue reservation.

## Ethics Statement

Written informed consent was obtained from the individual(s) for the publication of any potentially identifiable images or data included in this article.

## Author Contributions

All authors listed have made a substantial, direct and intellectual contribution to the work, and approved it for publication.

## Conflict of Interest

The authors declare that the research was conducted in the absence of any commercial or financial relationships that could be construed as a potential conflict of interest.

## Supplementary Material

Supplementary Figure 1 The verification of the down-regulation of ADAMTS1 in granulosa cells. The verification of the down-regulation of ADAMTS1 in granulosa cells by real-time PCR and western blot analysis **(A,B)**. si-Ctrl was used as control, ^****^ indicates *p* < 0.0001.

Supplementary Figure 2 The negative control of immunofluorescence in granulosa cells. The negative control was stained with rabbit IgG instead of ADAMTS1 primary antibody **(A)**. The negative control was stained with 5% BSA **(B)**. Blue, DAPI indicates the nuclear localization signal. Bar = 20 μm.

Supplementary Figure 3 The negative control of immunofluorescence in granulosa cells. The negative control was stained with mouse IgG instead of Ki-67 primary antibody **(A)**. The negative control was stained with 5% BSA **(B)**. Blue, DAPI indicates the nuclear localization signal. Bar = 20 μm.

Supplementary Figure 4 The negative control of immunofluorescence in embryos. The negative control was stained with rabbit IgG instead of ADAMTS1 primary antibody **(A)**. The negative control was stained with 5% BSA **(B)**. Blue, DAPI indicates the nuclear localization signal. Bar = 50 μm.
