# Supplementary figures and images for: Expression Level of ADAMTS1 in Granulosa Cells of PCOS Patients Is Related to Granulosa Cell Function, Oocyte Quality, and Embryo Development
Source: Front Cell Dev Biol. 2021 Apr 12;9:647522. doi: 10.3389/fcell.2021.647522 (PMC8075003; doi:10.3389/fcell.2021.647522)

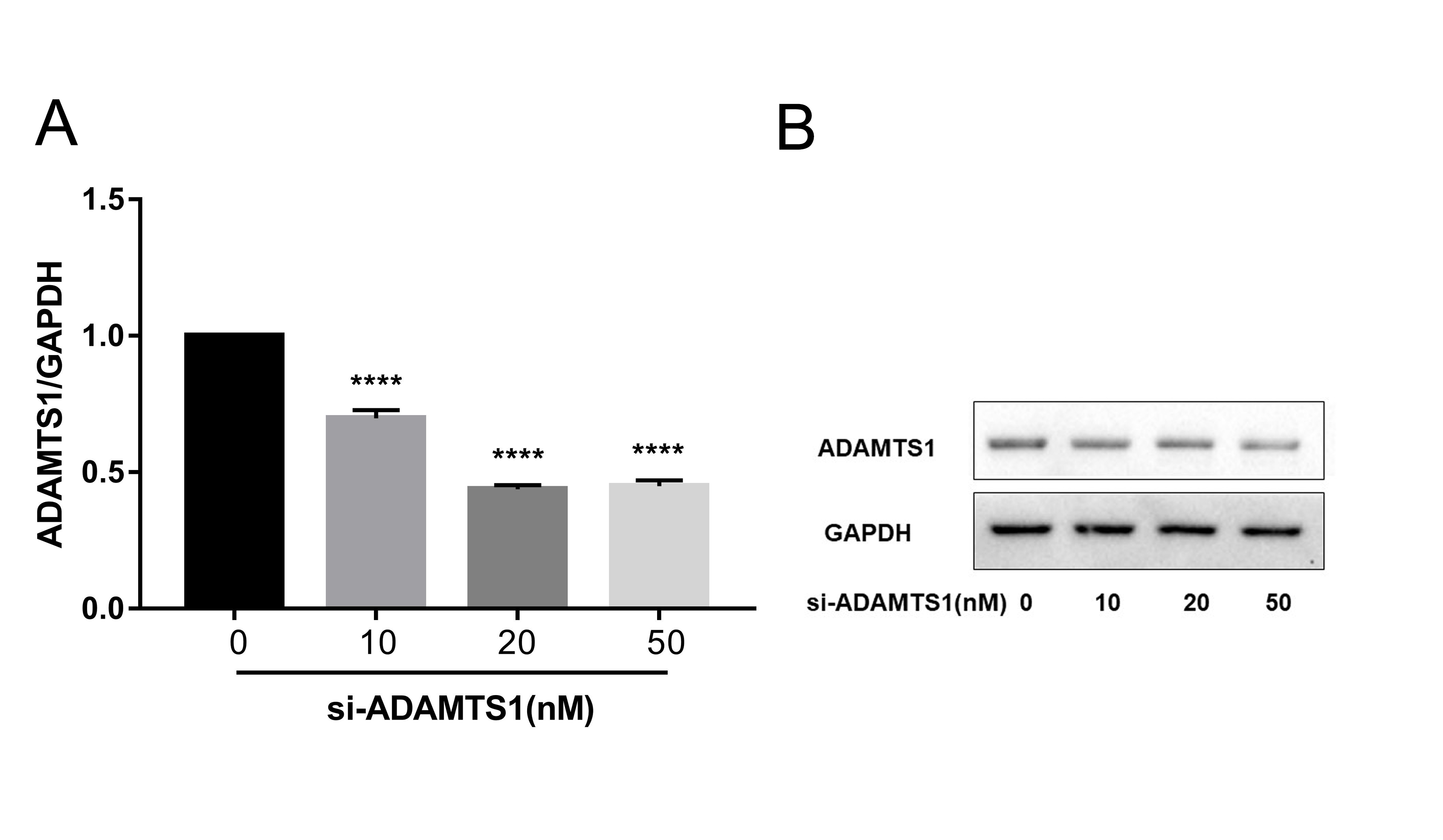

Supplement: Supplementary Figure 1 — The verification of the down-regulation of ADAMTS1 in granulosa cells. The verification of the down-regulation of ADAMTS1 in granulosa cells by real-time PCR and western blot analysis (A,B). si-Ctrl was used as control, **** indicates p < 0.0001. [file Image_1.TIF]

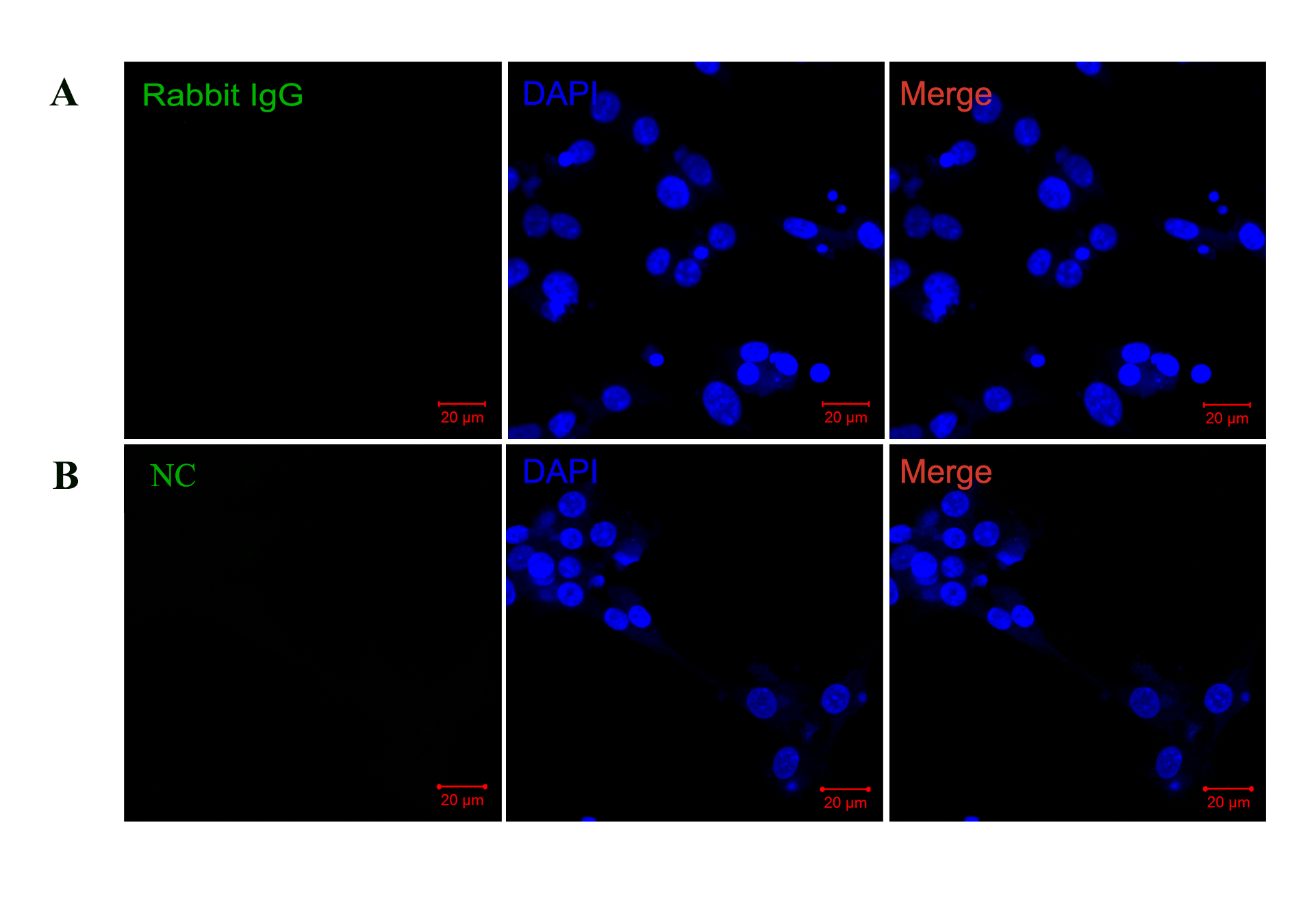

Supplement: Supplementary Figure 2 — The negative control of immunofluorescence in granulosa cells. The negative control was stained with rabbit IgG instead of ADAMTS1 primary antibody (A). The negative control was stained with 5% BSA (B). Blue, DAPI indicates the nuclear localization signal. Bar = 20 μm. [file Image_2.TIF]

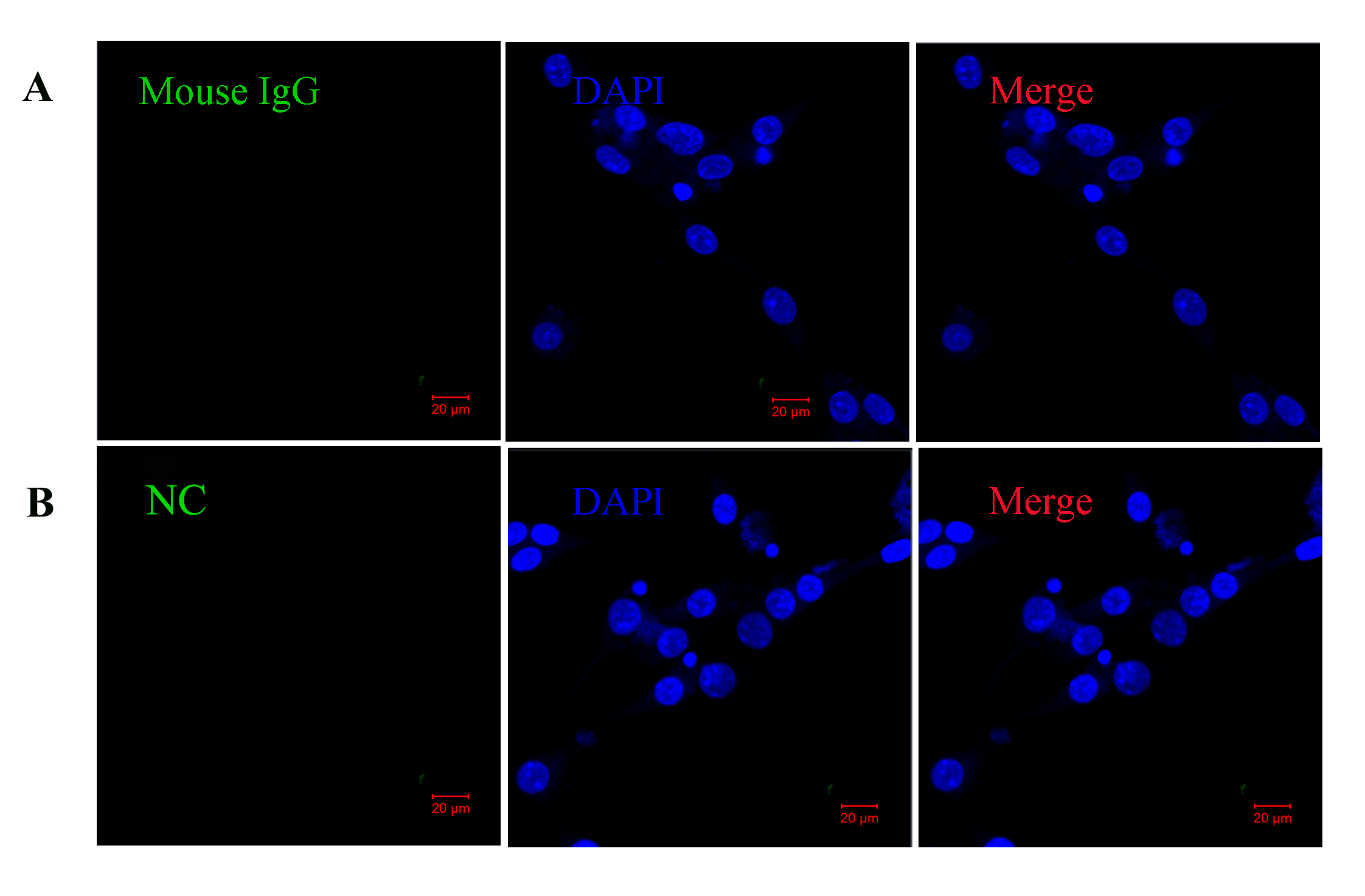

Supplement: Supplementary Figure 3 — The negative control of immunofluorescence in granulosa cells. The negative control was stained with mouse IgG instead of Ki-67 primary antibody (A). The negative control was stained with 5% BSA (B). Blue, DAPI indicates the nuclear localization signal. Bar = 20 μm. [file Image_3.TIF]

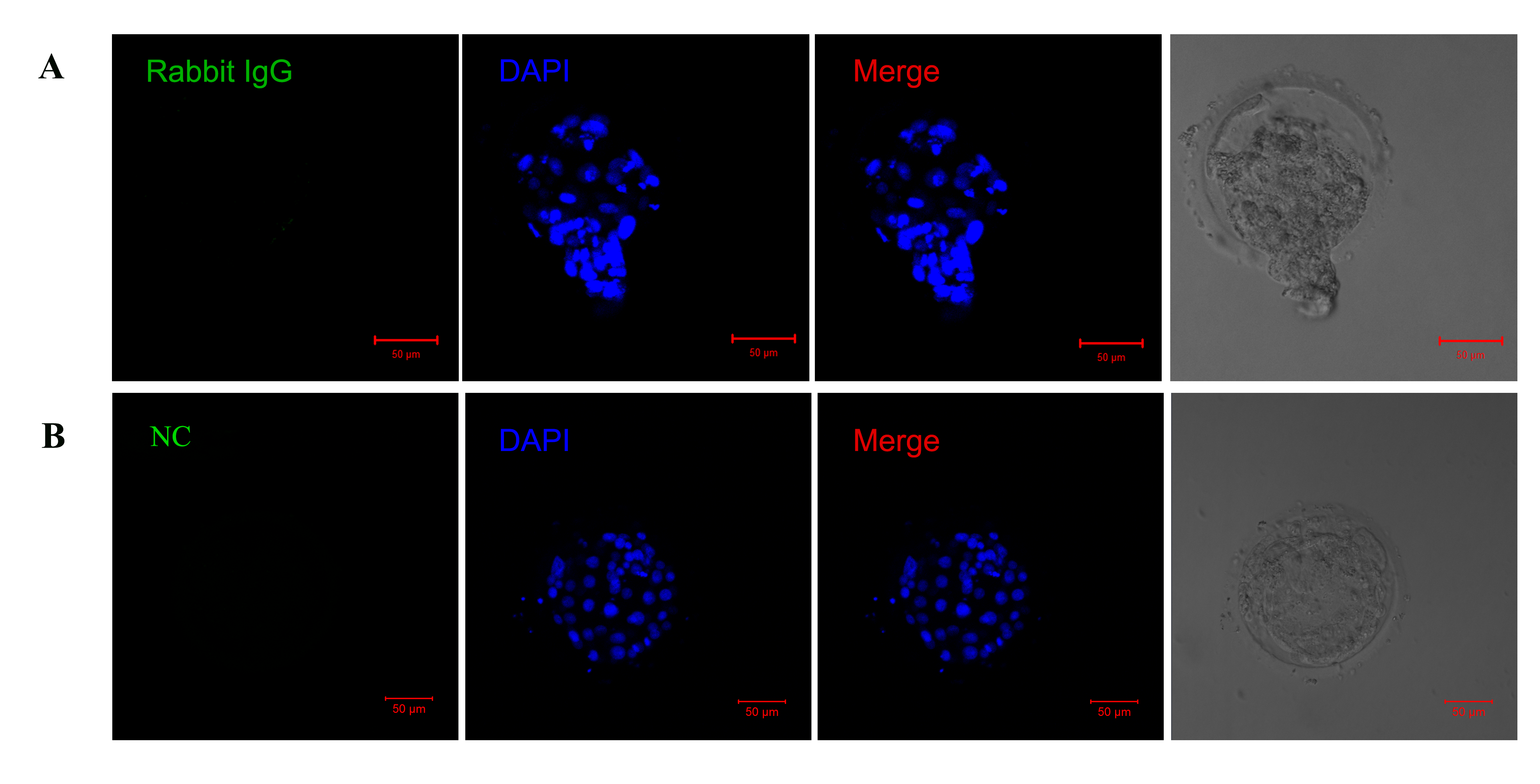

Supplement: Supplementary Figure 4 — The negative control of immunofluorescence in embryos. The negative control was stained with rabbit IgG instead of ADAMTS1 primary antibody (A). The negative control was stained with 5% BSA (B). Blue, DAPI indicates the nuclear localization signal. Bar = 50 μm. [file Image_4.TIF]
