# Supplementary material for: Expression Level of ADAMTS1 in Granulosa Cells of PCOS Patients Is Related to Granulosa Cell Function, Oocyte Quality, and Embryo Development
Source: Front Cell Dev Biol. 2021 Apr 12;9:647522. doi: 10.3389/fcell.2021.647522 (PMC8075003; doi:10.3389/fcell.2021.647522)
Supplement: Supplementary file 5 [file Table_1.doc]

**Supplementary Table 1. Primers used for real-time PCR**

| Gene | Forward primer (5’-3’) | Reverse primer (5’-3’) |
| --- | --- | --- |
| ADAMTS1 | GGACAGGTGCAAGCTCATCTG | TCTACAACCTTGGGCTGCAAA |
| GAPDH | GACAGTCAGCCGCATCTTCT | GCGCCCAATACGACCAAATC |
| Bcl2 | CATGTGTGTGGAGAGCGTCAA | GCCGGTTCAGGTACTCAGTCA |
| Bcl-XL | TCCTTGTCTACGCTTTCCACG | GGTCGCATTGTGGCCTTT |
| Bax | TGCCTCAGGATGCGTCCACCAA | CCCCAGTTGAAGTTGCCGTCAG |
| AREG | TGGACCTCAATGACACCTACTCTG | GGGCTTAACTACCTGTTCAACTCTG |
| VCAN1 | TGAGAACCCTGTATCGTTTTGAGA | CTGAATCTATTGGATGACCAATTACAC |
| HAS2 | GGTCGTCTCAAATTCATCTGATCTC | GGATACATAGAAACCTCTCACAATGC |
| PTX3 | ATTCAGAGGAAGGGCTCACA | TGCTCCTCCGGTCTCTCTTA |
| PAF | CACAAGACGGCACCCTATGT | GGCCGAAGGAGACACAATCA |
| HLA-G  LIF  COX2  THBS1  StAR  CYP11A1  CYP19A1 | CTGGTTGTCCTTGCAGCTGTAG  TGAACCAGATCAGGAGCCAACT  GCTCAAACATGATGTTTGCATTC  TGTGAAAAGATGGAGAATGCTG  AAACTTACGTGGCTACTCAGCATC  CAGGAGGGGTGGACACGAC  GGTCACCACGTTTCTCTGCT | CCTCCTTTTCAATCTGAGCTCTTC  CCACATAGCTTGTCCAGGTTGTT  GCTGGCCCTCGCTTATGA  TTGTGGCCAATGTAGTTAGTGC  GACCTGGTTGATGATGCTCTTG  AGGTTGCGTGCCATCTCATAC  GCAAGCTCTCCTCATCAAACCA |
